# Safety and efficacy of cytotoxic chemotherapy in hepatocellular carcinoma after first-line treatment with sorafenib

**DOI:** 10.1186/s12885-018-5173-0

**Published:** 2018-12-13

**Authors:** Leonardo Gomes da Fonseca, Guilherme Nader Marta, Maria Ignez Freitas Melro Braghiroli, Aline Lopes Chagas, Flair Jose Carrilho, Paulo Marcelo Hoff, Jorge Sabbaga

**Affiliations:** 1 Instituto do Cancer do Estado de São Paulo, Faculdade de Medicina da Universidade de São Paulo, Av Dr Arnaldo, 251, São Paulo, ZIP code: 01246-000 Brazil; 2 São Paulo Clínicas Liver Cancer Group, Instituto do Câncer do Estado de São Paulo – Hospital das Clínicas Complex, Department of Gastroenterology, University of São Paulo School of Medicine, São Paulo, Brazil

**Keywords:** Hepatocellular carcinoma, Sorafenib, Cytotoxic chemotherapy, Survival rate

## Abstract

**Background:**

Before the targeted therapies era, cytotoxic chemotherapy (CCT) was an option for advanced hepatocellular carcinoma (HCC), even with the lack of supporting evidence. Since the last decade, sorafenib has been established as the first-line therapy. Although new agents are being incorporated, CCT is still considered in regions where new drugs are not available or for patients who progressed through the approved therapies and remain in good clinical condition. We aimed to describe our experience regarding the use of CCT as second-line treatment after sorafenib.

**Methods:**

A database of 273 patients was evaluated. Patients that received CCT after sorafenib progression were selected for the analysis. Descriptive statistics was used for categorical and continue variables. Median survival was estimated with Kaplan-Meier curves. Variables were found to be significant if the two-sided p value was ≤ 0.05 on multivariate testing using the Cox regression model.

**Results:**

Forty-five patients received CCT; 33 (73.3%) had Child-Pugh classification A, and 34 (75.6%) had stage C according to the Barcelona Clinic Liver Cancer (BCLC) staging system. The most used regimen was doxorubicin in 25 patients (55.6%). Median overall survival (OS) was 8.05 months (95% confidence interval [CI] 2.73 – 9.88 months). The 6-month and 1-year survival probability was 52.4% and 27.36%, respectively. Eastern Cooperative Oncology Group performance status (ECOG PS) 0–1 and disease control with sorafenib was independently associated with better OS in patients treated with CCT. Any-grade toxicities were observed in 82.2% and grade 3–4 in 44.4% of the patients.

**Conclusion:**

In accordance with previous studies, CCT had a notable rate of adverse events. The poor prognosis of this cohort suggests that CCT may not alter the natural history of HCC after sorafenib progression.

## Background

Hepatocellular carcinoma (HCC) is the sixth most common neoplasm and the third leading cause of cancer-related death [1]. This high rate of mortality is mainly associated with the coexistence of underlying cirrhosis and the considerable proportion of patients diagnosed in advanced stage [2].

For patients in advanced stage and for those who are refractory or have contraindications to locoregional therapies, systemic treatment is recommended. Sorafenib, a multi-kinase inhibitor, showed consistent overall survival (OS) improvement in two phase III placebo-controlled trials and is considered the standard first-line therapy [3, 4]. Lenvatinib was shown to be non-inferior to sorafenib in a phase III non-inferiority trial [5]. In the second-line setting, regorafenib improved OS over placebo in patients who were tolerant and progressed on sorafenib [6]. Cabozantinib was also shown to be superior to placebo in terms of OS after at least one previous systemic therapy, including sorafenib, and can be an alternative in the second-line [7].

Before the targeted therapies era, cytotoxic chemotherapy (CCT) was considered a treatment option for advanced HCC. Indeed, in regions where the new agents are not available or for patients who progressed after the available therapies and remain in good clinical condition, CCT is still being considered albeit there is no high level of evidence.

Historically, doxorubicin was widely adopted in clinical practice although there was no proven benefit in terms of OS and safety [8]. Likewise, polychemotherapy with the PIAF regimen (cisplatin, interferon, doxorubicin and 5-fluorouracil) did not show significant survival benefit over doxorubicin and was associated with increased treatment-related toxicities [9]. In a phase III trial conducted in Asia that compared FOLFOX4 with doxorubicin, the median progression-free survival (PFS) favoured FOLFOX4, but there was no difference in OS between the two arms [10].

Even though the published results on CCT for HCC are disappointing, there are no randomised prospective trials that addressed a head-to-head comparison between CCT and sorafenib in the first-line setting or with other drugs as second-line therapy. Evolving therapies for HCC, such as immunotherapies and new target agents, will probably be incorporated into the treatment armamentarium in the near future. In this context, the role of CCT should be reviewed. We aimed to evaluate our single-centre experience regarding the safety and efficacy of CCT as second-line treatment in patients with HCC after sorafenib.

## Methods

### Patients and methods

A database of 273 consecutive HCC patients treated with sorafenib at Instituto do Cancer do Estado de São Paulo, Universidade de São Paulo, Brazil, from July 2009 to January 2017 was retrospectively evaluated after approval by the local ethics committee. All patients met diagnostic criteria for HCC based on radiological and/or histological findings [11].

From this dataset, we selected the patients that had received sorafenib as initial systemic treatment and were treated with CCT regimens, according to physician choice and local availability, after sorafenib discontinuation.

Relevant data from the clinical records were collected, including age, gender, Eastern Cooperative Oncology Group performance status (ECOG PS), pre-existing hepatopathy, Child-Pugh score, extrahepatic spread, vascular invasion, serum laboratory findings (alpha-fetoprotein, bilirubin, alkaline phosphatase and albumin), prior treatments for HCC, sorafenib treatment duration, CCT regimen, median CCT treatment duration, toxicity and OS. Outcome data were last updated on November 27, 2017.

### Treatment and assessment

The chemotherapy regimens used were: weekly bolus 5-fluorouracil (5-FU) 370 mg/m^2^ plus leucovorin 50 mg/m^2^ [12]; weekly oxaliplatin 85 mg/m^2^ at weeks 1, 3 and 5, weekly bolus 5-FU 500 mg/m^2^ with leucovorin 20 mg/m at weeks 1, 2, 3, 4, 5 and 6, then every 8 weeks (mFLOX) [13]; oxaliplatin 130 mg/m^2^ on day 1 plus capecitabine 1,000 mg/m^2^ twice daily on days 1 to 14, then every 3 weeks (CAPOX) [14]; doxorubicin 60–75 mg/m^2^ every 3 weeks [8]; gemcitabine 1,000 mg/m^2^ and oxaliplatin 85 mg/m^2^ every 2 weeks (GEMOX) [15] and capecitabine 1,250 mg/m^2^ twice daily for 2 weeks every 21 days and interferon (IFN)- alpha 3 million U three times a week. Usual dose reductions were done for managing adverse events depending on their type and severity. Treatment was continued until evidence of disease progression, unacceptable adverse event or death. The follow-up consisted of regular physical examination, laboratory assessment every 3–4 weeks and image studies (computed tomography or magnetic resonance) every 8–12 weeks. Assessment of response was based on the modified Response Evaluation Criteria in Solid Tumours [16]. OS was defined as the time from start of the CCT regimen treatment until death.

### Statistical analyses

Data were evaluated using SPSS software version 23.0 (SPSS Chicago, IL). Continuous variables were expressed as means and ranges. Categorical variables were expressed as frequencies and compared using the chi-square test. We analysed pre-treatment clinical and laboratory markers potentially associated with outcome. Kaplan-Meier curves comparing OS and 95% confidence interval (95%CI) between groups were constructed, and log-rank testing was used for comparisons in univariate analysis. Variables were found to be significant if the two-sided p value was ≤ 0.05 on multivariate testing using a Cox regression test.

## Results

### Baseline characteristics

Between July 2009 and January 2017, a total of 273 patients who received sorafenib as first-line therapy for HCC were enrolled in this analysis. Of the 273 patients, 33 patients were excluded due to lack of sufficient information in data records. From the 240 patients included, 51 (18.7%) received a second-line systemic therapy, of which 45 (16.4%) received CCT and 6 were included in clinical trials.

Median age of the CCT group was 61.7 years (range 18.9–76.6 years), the majority of patients were male (75.6%), 41 (91.1%) had ECOG PS 0–1, 33 (73.3%) had Child-Pugh classification A and 34 (75.6%) had stage C according to the Barcelona Clinic Liver Cancer (BCLC) staging system. Locoregional treatments had been previously delivered to 29 (64.4%) patients. The median duration of sorafenib treatment was 45 days (range 10–228 days). The most common reason for sorafenib discontinuation was progressive disease in 40 (88.9%) patients, while 4 (8.9%) patients discontinued sorafenib due to intolerance and 1 (2.2%) patient due to liver function deterioration. Doxorubicin was the regimen received by 25 (55.6%) patients, while the combination of fluoropyridine and oxaliplatin was received by 9 (20%) patients. The demographic and treatment characteristics are presented in Table 1.

**Table 1:** Baseline characteristics of the patients treated with chemotherapy

Baseline characteristics	*n*=45
Gender, n (%)	
Male	31 (75.6)
Female	14 (24.4)
Age, median years (min-max)	61.7 (18.9-76.6)
ECOG PS, n(%)	
0-1	41 (91.1)
2-3	04 (8.9)
Child-Pugh classification, n (%)	
A	33 (73.3)
B	11 (24.4)
C	1 (2.2)
BCLC stage, n(%)	
B	11 (24.4)
C	34 (75.6)
Chronic Liver Disease Etiology, n(%)	
HCV	20 (44.4)
HBV	9 (20.4)
Alcohol	8 (18.2)
NASH	3 (6.9)
Hemochromatosis	2 (4.5)
Auto-immune Hepatitis	2 (4.6)
Cryptogenic	1 (2.3)
Previous treatment, n (%)^a^	
Resection	7 (15.6)
RFA	2 (4.4)
Transplantation	3 (6.7)
TACE	18 (40)
None	16 (35.6)
Extrahepatic spread, n(%)	30 (66.7)
Vascular invasion, n(%)	13 (28.9)
CCT regimen (%)	
Doxorubicin	25 (55.6)
mFLOX/CapOx	9 (20)
5FU	8 (17.8)
GemOx	2 (4.4)
Capecitabine plus IFN	1 (2.2)
Ascites (%)	4 (8.9%)
Alpha fetoprotein, median ng/ml (IQI)	15514.4 (6683.5-24345.5)
Bilirubin median mg/dl (IQI)	1.27 (0.94 – 1.6)

ECOG PS: Eastern Cooperative Oncology Group performance status; BCLC: Barceona Clinic Liver Cancer; HBV: Hepatitis B virus; HCV: Hepatitis C virus; NASH: Non-alcoholic steatohepatitis; RFA: radiofrequency ablation; TACE: transarterial chemoembolization; CCT: cytotoxic chemotherapy; mFLOX: modified 5-fluorouracil and oxaliplatin combination; CapOx: capecitabine and oxaliplatin combination; 5FU: 5-fluorouracil; GemOX: gencitabibe and oxaliplatin combination; IFN: interferon; IQI: interquartile interval. ^a^some patients may have been treated with more than one previous treatment modality.

### Overall survival and response rate

**Fig. 1:**
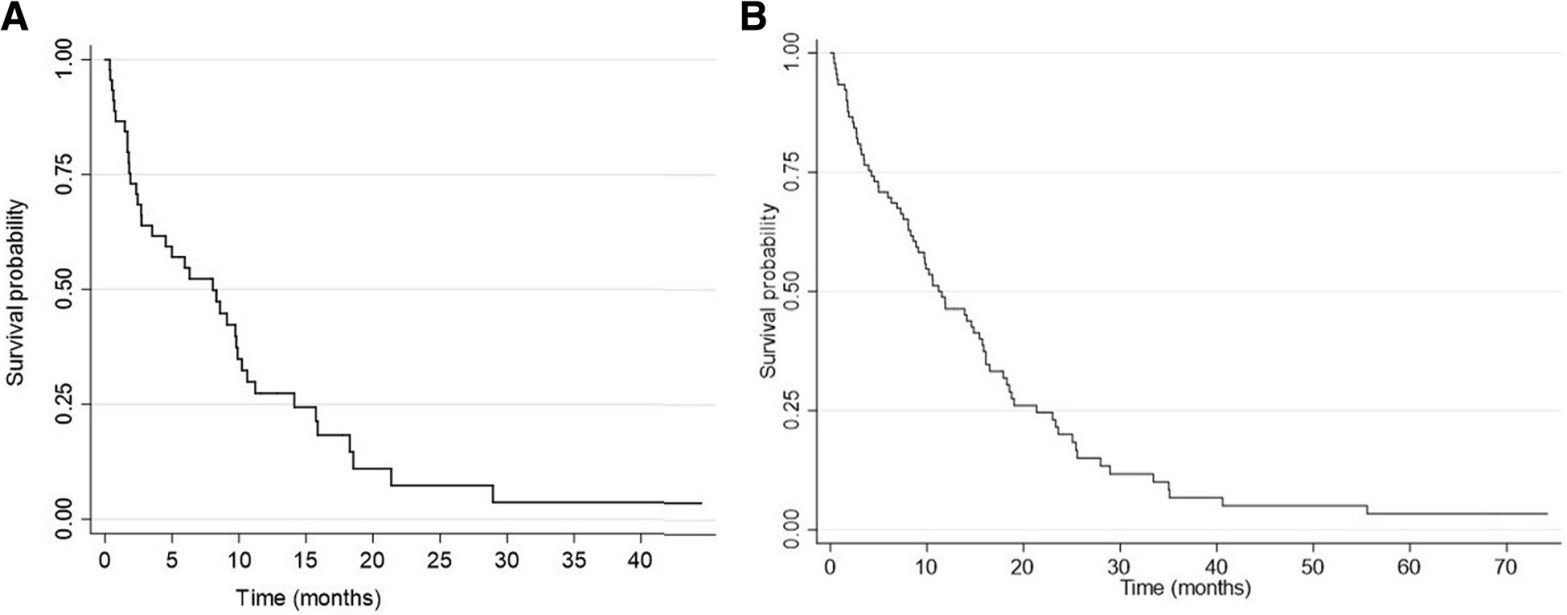
Survival curves of the patients treated with chemotherapy. **a** shows the survival curve from the start of chemotherapy until death. **b** represent the survival curve from the start of first-line sorafenib (sorafenib + chemotherapy) until death

At the time of the final analysis, 38 (84.4%) patients had died and 7 (15.6%) patients were still alive. The median follow-up was 6.31 months (95%CI 0.53 – 21.62 months). The median OS from the start of CCT until death was 8.05 months (95%CI 2.73 – 9.88 months) (Fig. 1a). The 6-month survival probability was 52.44% (95%CI 36.83 – 65.89%) and 1-year survival probability was 27.36% (95%CI 14.90 – 41.48%) from the start of CCT treatment. From the start of first-line sorafenib until death (sequence of sorafenib + CCT), the median OS was 15.86 months (95%CI 11.72 – 22.99 months) (Fig. 1b). There was no OS difference between CCT regimens (Table 2).

**Table 2:** Overall survival according to the chemotherapy regimen

Chemotherapy regimen	N	Median months	95%CI months
Doxorubicin	25	8.05	2.33-10.22
mFLOX/CapOx	09	8.57	0.62-NA
GemOx	02	3.51	3.51-NA
Capecitabine plus IFN	01	NA	NA
5-Fluorouracil	08	2.73	0.36-NA
Total	45	8.05	2.73-9.89

95%CI: 95% confidence interval; mFLOX: modified 5-fluorouracil and oxaliplatin combination; CapOx: capecitabine and oxaliplatin combination; gencitabibe and oxaliplatin combination; IFN: interferon.

Thirty-two patients had available radiological responses: partial response was reported for 2 (4.4%) patients, 6 (13.3%) patients had stable disease and 24 (53.3%) patients had progressive disease. The reason for discontinuation of CCT was unacceptable toxicity in 20 (44.4%) patients, progressive disease in 17 (37.8%) patients and liver function decompensation in 3 (6.7%) patients. Five patients were still on treatment with CCT.

The patients with BCLC C stage and Child-Pugh A (n=24) presented median OS of 8.05 months (95%CI 2.69-15.74) while those with Child-Pugh B (n=9) presented median OS of 1.91 months (95%CI 0.36-10.62). Considering the patients with BCLC B and Child-Pugh A (n=9), the median OS was 9.10 months (95%CI 1.49-9.88).

### Safety

Thirty-seven (82.2%) patients presented any-grade adverse events, including 20 (44.4%) patients with grade 3–4 events. The most frequent adverse events were nausea/vomiting in 16 (36%) patients and asthenia in 12 (27%) patients. The most frequent grade 3–4 adverse events were nausea/vomiting in 9 (20%) patients and myelotoxicity in 8 (18%) patients. No deaths related to the treatment were reported. Table 3 lists the adverse events presented.

**Table 3:** Rate of treatment-related adverse events

Adverse event	*N*= 45
Mielotoxicity	
Grade 1-2, n (%)	1(2)
Grade 3-4, n (%)	8(18)
Nauseas/Vomiting	
Grade 1-2, n (%)	7(16)
Grade 3-4, n (%)	9(20)
Hepatitis	
Grade 1-2, n (%)	2(4)
Grade 3-4, n (%)	3(7)
Nephrotoxicity	
Grade 1-2, n (%)	1(2)
Grade 3-4, n (%)	2(4)
Neuropathy	
Grade 1-2, n (%)	7(16)
Grade 3-4, n (%)	5(11)
Asthenia	
Grade 1-2, n (%)	7(16)
Grade 3-4, n (%)	5(11)
Diarrhea	
Grade 1-2, n (%)	3(7)
Grade 3-4, n (%)	2(4)
Mucositis	
Grade 1-2, n (%)	2 (4)
Grade 3-4, n (%)	1 (2)
Cardiotoxicity	
Grade 1-2, n (%)	1 (2)
Grade 3-4, n (%)	1 (2)

### Prognostic factors

**Fig. 2:**
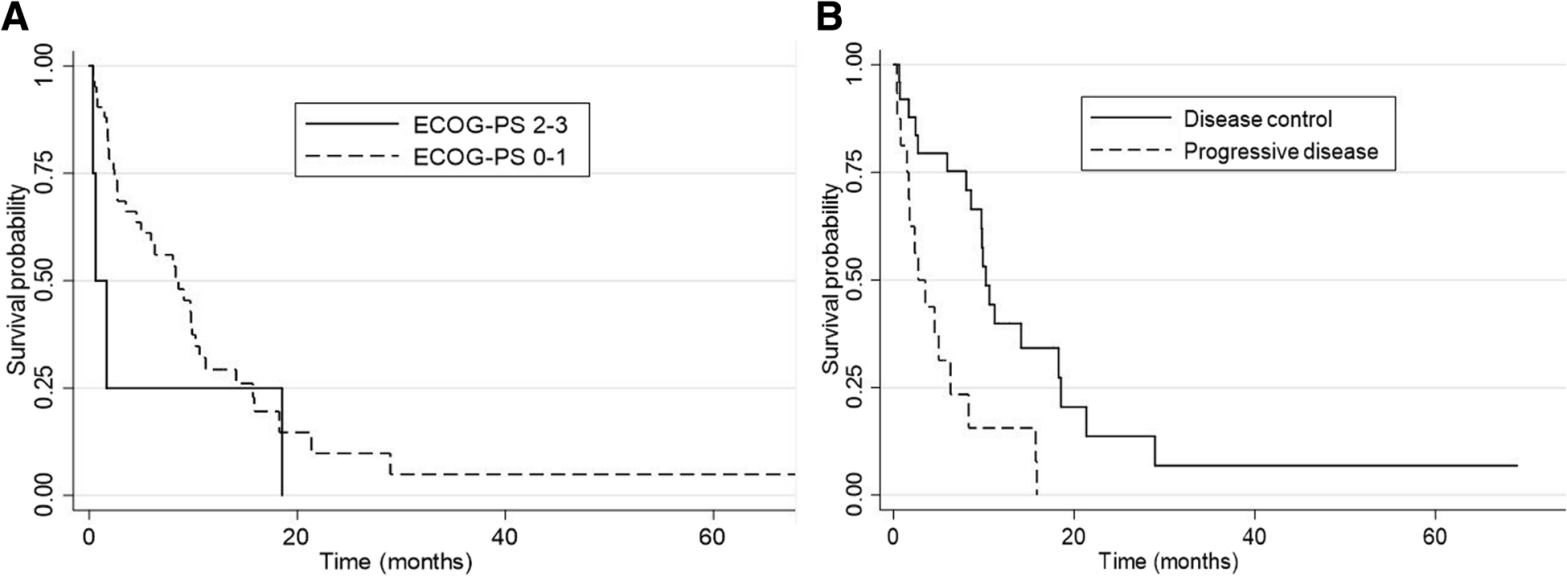
Survival curves representing prognostic factors associated with better survival in patients treated with chemotherapy. **a** shows the better survival for patients with ECOG-PS 0-1 vs ECOG-PS 2-3 (*p*=0.007). **b** shows that patients who presented disease control with first-line sorafenib had better survival versus those with progressive disease with sorafenib. (*p*=0.003)

We performed univariate analysis to identify potential prognostic factors in the cohort of patients submitted to CCT. Child-Pugh classification A at the start of CCT (versus B–C), ECOG PS 0–1 at the start of CCT (versus 2–3) and disease control (stable disease + partial response) versus progressive disease as best responses while on first-line sorafenib showed significant better survival and were further evaluated in a multivariate model. In the multivariate analysis, only ECOG PS 0–1 at the start of CCT (p = 0.007) and disease control while on first-line sorafenib (p = 0.003) were independently associated with better survival (Table 4 and Fig. 2).

**Table 4:** Prognostic factors in patients treated with chemotherapy

Prognostic factors	Median OS months (95%CI)	p univariate	p multivariate
Child-Pugh classification			
A	8.31 (2.72-9.88)	0.0251	0.524
B-C	3.51 (0.52-14.12)		
ECOG performance status			
0-1	9.36 (2.79 – 18.26)	<0.001	0.007
2-3	1.08 (0.36- 2.01)		
Best response to sorafenib			
Progressive disease	2.73 (1.48-6.31)	0.0035	0.003
Disease control	9.89 (8.04-14.13)		

OS: overall survival; 95%CI: 95% confidence interval; ECOG: Eastern Cooperative Oncology Group

## Discussion

In the present study, we described our experience with CCT in patients with HCC after discontinuation of sorafenib. We found a high rate of any-grade adverse events, including a notable incidence of grade 3–4 adverse events, and an OS of approximately 8 months, denoting a poor prognosis in this cohort of HCC patients treated with CCT.

The systemic therapy for advanced HCC has changed since the results of the SHARP [4] and ASIA-PACIFIC trials [3] in which sorafenib delayed tumour progression, improved survival and became the first approved therapy for unresectable HCC. In the following years after these trials, many agents have been tested with disappointing results both in the first- and second-line setting [17–19].

There have been many attempts to implement CCT as an option for systemic treatment of HCC. However, the published data failed to prove a convincing benefit. CCT was associated with low response rates, poor OS and a significant rate of adverse events. Currently, the main guidelines do not support the use of CCT in HCC [16, 20].

A prospective randomised trial that assessed the efficacy of doxorubicin over no antitumour therapy reported an OS of 10.6 weeks and a response rate of less than 10% of the patients. In this trial, doxorubicin was associated with fatal complications in 25% of the patients, including septicaemia and cardiotoxicity [8].

Another prospective study compared doxorubicin and the PIAF regimen. There was no statistical difference in terms of OS between the two arms and a high rate of treatment-related toxicity was observed in patients treated with PIAF [9]. Additionally, FOLFOX4 did not show benefit in OS over doxorubicin in a phase III trial [10]. Finally, some observational studies evaluated GEMOX. The results often showed an acceptable toxicity profile, but the efficacy, in terms of prolonging survival, was not encouraging [15, 21].

The combination of sorafenib and doxorubicin was also evaluated in a double-blind phase II study in which sorafenib plus doxorubicin resulted in prolonged PFS (6.0 *vs.* 2.7 months, *p* = 0.006) and OS (13.7 *vs.* 6.5 months, *p* = 0.006) compared with doxorubicin monotherapy [22]. Based on the promising results of this phase II study, Cancer and Leukaemia Group B (CALGB) conducted a phase III trial randomising sorafenib plus doxorubicin compared to sorafenib alone. However, the preliminary report presented in 2016 demonstrated that the combination of sorafenib and doxorubicin was associated with shorter OS (9.3 *vs.* 10.5 months) and higher toxicity than sorafenib alone [23].

This disappointing results with CCT in HCC may be explained by the underlying liver cirrhosis, which limits the clinical management and imposes impairments in the drug metabolisation. This fact supports the high rate of grade 3–4 adverse events observed in our cohort and in the previously mentioned studies.

Recently, the multi-kinase inhibitor regorafenib improved survival in a phase III placebo-controlled trial in the second-line setting. Those patients treated with this agent had a median OS of 10.6 months versus 7.8 months for those randomised to the placebo arm. Based on these results, regorafenib was recommended as the first second-line therapy in HCC [6]. In a recently published randomised trial, cabozantinib was also superior to placebo in prolonging survival in patients with HCC, with median OS of 10.2 versus 8.0 months [7]. Although it is not fair to perform interstudy comparisons, these two second-line trials indicated that the OS for the placebo arm of selected patients was around 8 months. Our cohort was composed of selected patients who were considered by the physician to be in good clinical condition to receive additional treatment after sorafenib. However, our cohort achieved a median OS that was numerically similar to that observed in the placebo arms of the trials that tested cabozantinib and regorafenib in the second-line [6, 7]. This result was consistent even with consider only the subgroup of our cohort with Child-Pugh A and BCLC C, that is the predominant subset of patients included in the mentioned second-line trials. Altogether, it suggests that CCT did not impact the natural history of HCC after sorafenib progression.

A limitation of our study is that we grouped different CCT regimens to evaluate outcomes and safety. The hypothesis that one of the regimens may be beneficial post-sorafenib cannot be determined owing to the small sample size of patients that received each regimen. However, even the regimens that were the most represented (mFLOX, CapOx and Doxorrubicin) did not show a median OS that suggested activity regarding the current scenario of second-line systemic therapies for HCC. Besides, previous published safety data of these regimens are not encouraging [8, 10].

The use of CCT in HCC was motivated mainly because of an historic lack of effective therapies rather than based on solid evidence. However, the context seems to be changing, as we have positive phase III results for regorafenib and cabozantinib [6, 7]. Additionally, new data on immunotherapy shows promising activity in HCC. The phase I/II trial CHECKMATE 040 reported a median OS of 15.0 months for patients treated with nivolumab after sorafenib [24]. Results of phase III trials of immunotherapy agents are being awaited, although the costs of these new therapies may delay their incorporation, especially in developing countries.

## Conclusion

In accordance to previous published results, our results demonstrated that CCT has a notable rate of adverse events and seems to have a low efficacy in patients with HCC.

## Abbreviations

5-FU: 5-fluorouracil
BCLC: Barcelona Clinic Liver Cancer
CALGB: Cancer and Leukaemia Group B
CAPOX: Oxaliplatin plus capecitabine regimen
CCT: Cytotoxic chemotherapy
ECOG PS: Eastern Cooperative Oncology Group performance status
GEMOX: Gemcitabine plus oxaliplatin regimen
HCC: Hepatocellular carcinoma
IFN: Interferon
mFLOX: Oxaliplatin plus 5-fluorouracil regimen
OS: Overall survival
PFS: Progression-free survival
